# Launching the ACL Innovation Lab: Demonstrating a Core Components Approach for Reducing Older Adult Falls

**DOI:** 10.1093/geroni/igaf122.1746

**Published:** 2025-12-31

**Authors:** Emily Nabors, Liz Noble, Matthew Smith

**Affiliations:** National Council on Aging, Arlington, Virginia, United States; Mission Measurement, Chicago, Illinois, United States; Texas A&M University, College Station, Texas, United States

## Abstract

Falls among older adults are common, and while evidence-based falls prevention programs are available, they can be challenging for organizations with limited capacity to offer and sustain, and they may not resonate with all populations. To address these challenges, the Administration for Community Living (ACL) funded the National Council on Aging to become the ACL Innovation Lab. In partnership with Impact Genome and several academic institutions, the Lab developed the first research-based falls prevention taxonomy to identify core components of interventions that address falls risk factors among older adults. A core components approach allows practitioners to adapt interventions to fit different populations or settings by integrating components that have shown relationships to positive outcomes in research. The Lab conducted a literature review on falls prevention interventions and coded the literature to draft the falls prevention taxonomy and its four frameworks of core intervention components, intended recipients, contextual elements, and outcomes. After applying the frameworks to the evidence base to build a coded and structured dataset, the Lab conducted a taxonomic meta-synthesis to identify core components based on their relationship to falls prevention outcomes. This session will define a core components approach and how this methodology can be applied to other aspects of gerontology. Presenters will introduce the core components approach and falls prevention taxonomy; describe how selected core components are being piloted in community-based settings to test the feasibility, flexibility, acceptability, and sustainability of this approach to reducing falls among older adults; and outline the research questions guiding this work.

